# First-Step PPG Signal Analysis for Evaluation of Stress Induced during Scanning in the Open-Air MRI Device †

**DOI:** 10.3390/s20123532

**Published:** 2020-06-22

**Authors:** Jiří Přibil, Anna Přibilová, Ivan Frollo

**Affiliations:** Institute of Measurement Science, Slovak Academy of Sciences, 841 04 Bratislava, Slovakia; Anna.Pribilova@savba.sk (A.P.); Ivan.Frollo@savba.sk (I.F.)

**Keywords:** acoustic noise, cardiovascular system parameters, magnetic resonance imaging, Oliva–Roztocil index, photoplethysmographic sensor

## Abstract

The paper describes first-step experiments with parallel measurement of cardiovascular parameters using a photoplethysmographic optical sensor and standard portable blood pressure monitors in different situations of body relaxation and stimulation. Changes in the human cardiovascular system are mainly manifested by differences in the Oliva–Roztocil index, the instantaneous heart rate, and variations in blood pressure. In the auxiliary experiments, different physiological and psychological stimuli were applied to test whether relaxation and activation phases produce different measured parameters suitable for further statistical analysis and processing. The principal investigation is aimed at analysis of vibration and acoustic noise impact on a physiological and psychological state of a person lying inside the low-field open-air magnetic resonance imager (MRI). The obtained results will be used to analyze, quantify, and suppress a possible stress factor that has an impact on the speech signal recorded during scanning in the MRI device in the research aimed at 3D modeling of the human vocal tract.

## 1. Introduction

At present, non-invasive, fast, and precise methods for investigation of a human body are increasingly used in clinical practice. The primary aim is to minimize the absorbed radiation during the examination in X-ray or microwave computed tomography (CT) scanners [1]. Application of other methods and devices working on different physical principles also brings unwanted negative physiological and psychological effects on examined persons, for example, exposition to noise and vibration can pose a health risk to humans [2]. This may be the case of the magnetic resonance imaging (MRI) approach where the negative effect is caused by exposition of a patient to vibration and acoustic noise produced by the gradient system of this type of a scan device. Principally, MRI scanners can be divided into two types—the open-air ones working with a weak magnetic field (up to 0.2 T) used mainly for scanning of peripheral parts of the human body (arm, leg, neck, and so on) and the whole-body ones working with stronger basic magnetic fields (at present, up to 9 T) for widespread usage in MR imaging. There exist complex studies dealing with description of measurement, analysis, and comparison of an acoustic noise and its properties during various pulse MR sequences at field strengths of 0.5, 1.0, 1.5, and 2.0 T [3], and acoustic noise levels for fast MR pulse sequences were explored in systems with field strengths ranging from 0.2 T to 3 T [4]. The whole-body MRI device may have a negative influence on an examined person lying inside the scan tube, especially in claustrophobic patients [5]. In all cases, the current negative consequences depend primarily on the acting intensity and exposure time, but they are strongly individual for every person.

The negative influence of the generated vibration and noise on a human body and psychic can be monitored by measuring the blood pressure (BP) and heart rate (HR) during MR scanning. The induced stress may be manifested by changes in the bloodstream or can also be detected in the simultaneously recorded speech signal [6,7]. In the present paper, we describe photoplethysmographic (PPG) measurement using an optical sensor for non-invasive pickup of vital functions of the vascular system from the skin [8,9] by detecting blood volume changes inside the tissue. Signal filtering and further processing yields a clean PPG waveform that is then used to determine an instantaneous heart rate and other specific parameters applicable in systems for medical assessment [10,11,12], and/or utilized for biometric authentication [13], among others. In addition, this type of a sensor enables measurement in the low magnetic field present in the scanning area of an MRI device. For purpose of this study, we are mostly oriented on the instantaneous heart rate and the parameter called the Oliva–Roztocil index (ORi), which is often successfully used in vascular damage analysis [14] and as a promising new way to quantify pain [15,16].

The motivation of our work was to detect and analyze the possible stress that has an impact on the speech signal recorded simultaneously for 3D modeling of the human vocal tract [17]. This stress causes tensions in vocal cords and negative modification of suprasegmental and spectral features of the speech signal, so it can bring about errors and inaccuracy in the calculation of 3D models of the human vocal tract. In correspondence with previous research [18,19,20], the main long-term aim of our investigations is to find a methodology and recommendations for the minimization of negative physiological and psychological stress as the secondary undesirable effect of scanning inside the low-field MRI device. The final task is to find proper settings of MR scan parameters (such as TE and TR times, type of sequence, number and thickness of slices, and so on) that minimize generated vibration and acoustic noise, decrease the stress factor induced on an examined person, and eventually increase the accuracy of the created 3D vocal tract models.

Our current work is based on a premise that changes in ORi as well as HR and BP parameters can be affected by different physiological as well as psychological stress factors, and can also be experimentally invoked by a proper stimulus. The group of physical stimuli includes any performed activity such as sport exercise, change in body position (from sitting to lying or vice versa), and so on. Drinking coffee, tea, alcoholic, or energetic drinks, among others, also falls into this group. However, the effect of caffeine on blood pressure is controversial [21]. Acute ingestion of caffeine elevates blood pressure, but chronic caffeine intake has less of an effect on blood pressure. Systolic and diastolic blood pressures increased by caffeine are not accompanied by altered heart rate. On the other hand, long-term intake of coffee decreases blood pressure in patients with mild hypertension, but acute intake of coffee has no effect on blood pressure in these patients. Caffeine elevates blood pressure in patients with coronary artery disease, but not in subjects without coronary artery disease [21]. When compared with caffeine, the effect of alcohol consumption is more distinct in the risk of hypertension and other cardiovascular diseases [22]. Nevertheless, similar physiological changes can be induced by psychological stimuli producing the stress effect on the human body. These stimuli are very individual and depend strongly on the age (first of all, for older persons) and the current physical and mental condition of the tested person. Generally, different persons react differently to music listening, film or TV watching, book reading, and so on. In addition to basic audio-visual stimuli, other basic external factors as vibration, noise, heat or cold, dry or humidity, and so on are perceived by humans and may affect heart parameters directly or indirectly.

The experimental measurements were realized in two basic parts: the initial/final one without any stimuli (further called Relax) and the second one with application of different types of physical/mental stimuli (further called Load). Three types of experiments were practically realized. The introductory measurements aimed to find whether the passive Relax and active Load phases produce different physiological parameters that would be separable for subsequent statistical analysis. The second preliminary experiments analyzed the situation of a person listening to the MRI noise through headphones in the Load phase while sitting at a table. In the frame of the main measuring experiments, the tested person lies in the scanning area of the MRI device. Here, we compare results from the active stimulation Load phases with an MR scan sequence running and the Relax phases without any scanning activity. Real-time recording of the PPG signal was accompanied with parallel measuring of the BP and HR by portable blood pressure monitors (BPMs). The obtained results were then statistically processed and visualized or presented in the numerical form.

## 2. PPG Signal and Its Basic Parameters

**Figure 1:**
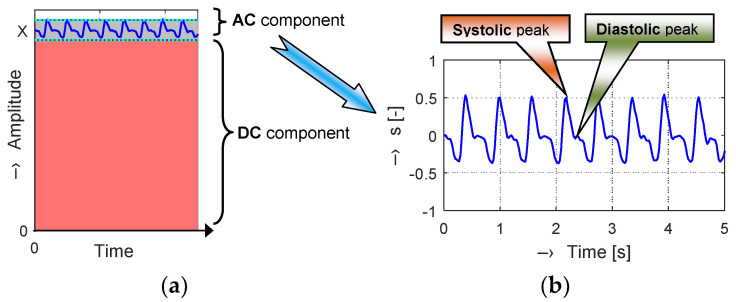
Principal structure of a photoplethysmographic (PPG) signal: (**a**) alternating current (AC) and direct current (DC) components superposition; (**b**) PPG volume pulse wave after signal processing (pre-filtering and DC component removal).

A great part of the PPG signal is composed of a direct current (DC) component corresponding to the whole blood volume of an examined tissue. The superimposed alternating current (AC) component follows the beating of the heart, so it also carries vital information including the heart rate. Its magnitude is much smaller (typically about 2% of the DC component), as can be seen in a principal diagram in Figure 1a. In each PPG cycle, two maxima are observed, representing systolic and diastolic peaks that provide valuable information about the cardiovascular system.

Depending on the shape and location of the PPG signal recording, it is possible to distinguish the so-called central and peripheral pulse wave. We can also see the peripheral pulse wave at the ends of fingers on each limb. In this case, optical sensors working in the transmission mode are usually used. Variations in the photodetector signal are related to changes in blood volume inside the tissue. The picked-up signal is then filtered and amplified to obtain a clean PPG waveform (see an example in Figure 1b) necessary to determine the parameters describing actual condition of the cardiovascular system of the tested person.

In practice, in addition to HR values, another two parameters are often calculated from the PPG signal. The first one is an index of blood supply to tissues (TPI)

(1) $$TPI=\frac{rel_{AC}}{rel_{DC}}\cdot \frac{1}{TD_{P-P}},$$

where the parameters *rel*_AC_ and *rel*_DC_ represent relative parts of AC and DC components of the PPG signal, and *TD*_P-P_ is the time duration between two consecutive systolic peaks. If the DC component cannot be measured or it is not present (using the reflective PPG sensor or owing to inherent PPG signal post-processing), the parameter of choice for PPG peripheral pulse wave analysis is the Oliva–Roztocil index. Several simulation and experimental studies have shown that ORi can also be used for the quantification of pain and/or stress, which is also our main area of interest. The special merit of ORi lies in its proposed normalization of the blood volume pulse (pulse amplitude and pulse length) as [14]

(2) $$ORi=\frac{W_{23}}{TD_{P-P}},$$

where the parameter *W*_23_ represents the width of the systolic pulse at the height of two-thirds from the basis (one-third from the top).

## 3. Processing of the PPG Signal and the Oliva–Roztocil Index Determination

**Figure 2:**
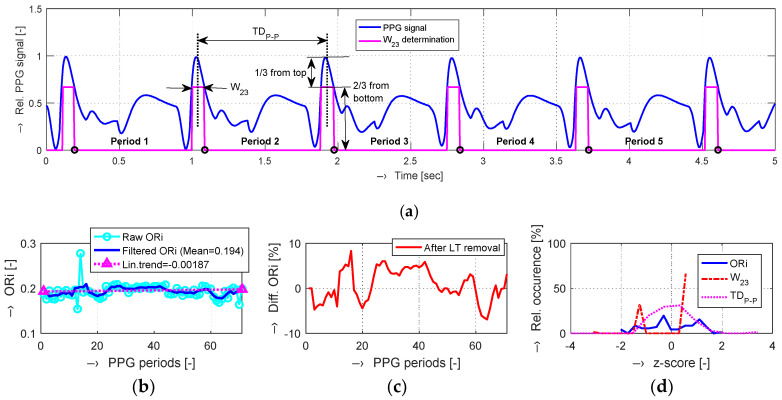
An example of PPG signal processing: (**a**) a 5 s region of interest (ROI) of a down-sampled signal after application of the absolute value operation (originally recorded at *f*_s_ = 2 kHz, total time duration was 72 s) with localized peaks and determined parameters *W*_23_ and *TD*_P-P_; (**b**) raw and filtered Oliva–Roztocil index (ORi) values with the finally calculated linear trend (LT) of the whole PPG signal record; (**c**) differential ORi after LT removal; (**d**) histograms of z-scores calculated from the ORi, *W*_23_, and *TD*_P-P_ values.

The PPG signal from an optical sensor after analog pre-treating was subsequently processed in the following steps:

- down-sampling the PPG signal originally sampled at *f*_s_ frequency by a factor of *Dns*: *f*_dns_ = *f*_s_/*Dns*;
- application of the absolute value operation on the down-sampled PPG signal with the output range of <0, 1> for further analysis;
- localization of the main PPG peak positions (affected by the systolic heart pulse), determination of maximum and minimum values;
- determination the *W*_23_ parameter—see an example for a selected region of interest (ROI) of 5 s in Figure 2a;
- calculation of *TD*_P-P_ distances between the localized systolic peaks in [s];
- calculation of the heart rate (HR) in [min^−1^] from *TD*_P-P_ as $HR=60/TD_{P-P}$;
- calculation of ORi values using (2);
- smoothing of ORi values by a three-point median filter and linear trend (LT) calculation for the whole time duration (all periods) of the processed PPG signal—see an example in Figure 2b;
- calculation of percentage of differential ORi values after LT removal—see Figure 2c;
- basic statistical analysis and histogram creation for further comparison—see Figure 2d.

## 4. Description of Performed Experiments

Practically, three types of measurements were performed:

1. Auxiliary experiments comprising the PPG signal recording in chosen different physiological as well as psychological activities: music/noise listening, drinking tea or coffee, some sporting physical activity, and so on, with the aim to test the extreme situations for further statistical analysis of obtained HR, BP, and ORi parameters. The main aim of these auxiliary experiments is to find whether the relaxation and activation phases produce different HR and ORi values determined from the PPG signal, and whether the obtained results are separable (significantly different) for further statistical analysis. These measurements were performed in normal room/laboratory conditions with a tested person sitting on a chair at a table.
2. Preliminary experiments consisting of a situation when the tested person sitting on a chair by a table hears the MRI noise through headphones in the *Load* phases. This noise was recorded from inside the MRI scanner during execution of the MR scan sequence having the most vibration energy (3D SSF with TE = 10 ms, TR = 40 ms, sagittal orientation, 3D-phases = 24, *N*_ACC_ = 4 [19,20]). In the silent, *Relax* phases, the normal room ambient noise is present without wearing headphones.
3. Main measuring experiments when the tested person lies in the MRI scanning area and his/her PPG signal is recorded simultaneously for further analysis and processing. In the active stimulation phases, the MR scan sequence is running, while in the *Relax* phases, a person is lying in a tomograph with no scan activity. Practical measurement experiments were realized on the MRI equipment E-scan Opera by Esaote S.p.A. [23] located at the Institute of Measurement Science in Bratislava. This open-air MRI device works with a stationary magnetic field of 0.178 T. For backward compatibility with our previous experiments [18,19,20], the high-resolution SE-HF scan sequence with TE = 18 ms, TR = 400 ms, and sagittal orientation was applied.

**Figure 3:**
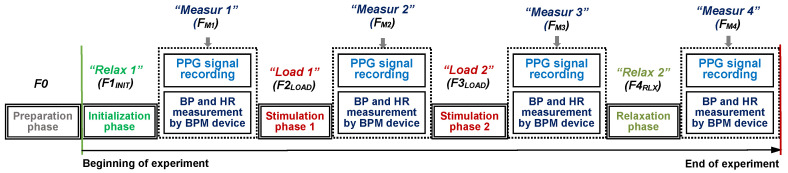
Principal measurement schedule applied in all performed experiments. BPM, blood pressure monitor; HR, heart rate.

Each individual experiment is divided into four phases—two Relax ones (without any physical or mental activity of a tested person) and two Load ones when a person is exposed by different audio-visual stimuli or he/she executes some physical activity. The time duration (*T*_DUR_) of each of these four phases is about 300 s. The experiment starts with the first initial Relax phase necessary for preparation of a person for the measurement (adaptation), further called F1_INIT_, and finishes with the second relaxation phase F4_RLX_—see the principal measurement schedule in Figure 3. The second and the third phases (F2_LOAD_ and F3_LOAD_) represent the active stimulation of the tested person. Each of these phases is followed by a shorter one (F_M1_,.., F_M4_) with simultaneous manual measurement of BP and HR parameters by a portable BPM device during the PPG signal recording (with *T*_DUR_ of about 60 s). Therefore, the total expected duration of the experiment is finally about 24 min. This time duration was chosen with the duration of the most used scan sequences applied in the investigated MRI device. The scanning times for the 3D and hi-res sequences are generally less than 15 min, typically about 3–5 min, depending on the chosen number and thickness of the slices [23]. In this way, the requirement was fulfilled that an exposition of the human organism and auditory system to the noise and vibration is not great, but the physiological effect is measurable and can be reliably detected. Before the measurement (in the preparation phase F0), the subject entering and positioning inside the MRI device, the PPG sensor adjustment, and the BMP pressure cuff wearing operations are carried out, but they are not counted in the final time of the experiment. This is important because, during the scanning process, no movements of the examined person are appropriate to obtain the final MR image(s) without any blurring effect. The experiment practically starts with the phase F1_INIT_ when the tested person is adapting after a change of his/her position from staying to lying and gets used to the environment of the MRI device scanning area.

In all cases, the PPG signal was recorded by an optical sensor HRM-2511E (Kyoto Electronic Co., China) operating in the transmission mode to pick up the blood variation in the finger tissue and outputting a digital pulse synchronous with the heartbeat. Such an optical sensor is suitable for usage in a magnetic field environment with radio frequency (RF) and electromagnetic disturbance that is principally presented in the scanning area of the MRI device [23]. The optical sensor practically consists of an infrared emitting diode as a source and an infrared phototransistor as a detector. The sensor body is constructed from a flexible silicone rubber material keeping the sensor adhered to the finger. The signal from the photo detector is pre-amplified and processed by the analog interface Easy Pulse Version 1.1 (ER-CDE10301E) (Embedded Lab, Williamsburg, VA, USA). This Easy Pulse V1.1 sensor module [24] contains a quad operational amplifier (OA) with rail-to-rail output capability for maximum signal swing from the photo detector. As this output is weak and noisy, amplifier and filter circuits are used to boost and clean the signal. First, it is passed through a passive RC high-pass filter (HPF) to block the DC component of the PPG signal with a cut-off frequency of about 0.5 Hz. The output from the HPF is passed through an OA-based active low-pass filter (LPF) with the cut-off frequency set to 3.4 Hz. Its output then passes to the second stage of a filter consisting of similar HPF and LPF blocks in a cascade connection. The signal amplified and filtered in these two steps is now fed to a third OA, which is configured as a non-inverting buffer with a unity gain [24]. The output of the buffer provides the required analog PPG signal. This signal is fed to the mixer Behringer XENYX Q802 and through its USB interface to the PC, where it is first sampled at *f*_s_ = 2 kHz and then downsampled. For processing of the PPG signals recorded in the frame of the auxiliary experiments, *Dns* = 13 (*f*_dns_ = 153.85 Hz) was used. On the other hand, *Dns* = 12 (*f*_dns_ = 166.67 Hz) was applied for analysis of the PPG signals originating from the preliminary and main experiments (inside the MRI device). After PPG wave recording, its amplitude is normalized to −16 dB by the software Sound Forge 9.0a. The implemented analog filters with a cut-off frequency below 50 Hz suppress the power-line frequency and its multiples in the signal chain. However, the problem of earth loops would not be solved completely if pieces of measurement equipment (mixing console, laptop) were connected to the mains earths at even slightly different potentials. There is also a possibility of interference or modulation of a PPG signal owing to galvanic connection through a supply of operational amplifiers used for pre-processing of an optical sensor signal having a relatively small amplitude. This problem was solved here by the USB connected battery-based 5 V power supply of the power bank AlzaPower Source 20,000 mAh Quick Charge 3.0 (Alza.cz, Prague, Czech Republic).

The choice of the optical sensor HRM-2511E working in the transmission mode together with the analog interface Easy Pulse was made for its successful application in the measurement that was already presented in [25]. Prior to this, we had also tested several PPG sensors based on a reflection principle, but the obtained results were unsatisfactory owing to not very good PPG signal purity and stability. In the frame of the mentioned previous research, we have also compared results of HR values measured by three tested BPMs with those determined from the PPG signal. The best results with minimal dispersion and approximately zero mean value of calculated relative differences were achieved by the automatic blood pressure monitor BP A150-30 AFIB by Microlife AG, (Swiss Corporation, Widnau/Switzerland). Hence, this type of BPM was used in the present experiments. The PPG signal was picked up from the opposite hand to prevent a possible negative influence of an inflated pressure cuff of BPM on a tested person’s blood system. For measurement in the weak magnetic field environment, inside the scanning area of the MRI scanner (within the main experiment), the metallic fastening buckle of the measuring arm cuff was substituted by a 3 mm Cu wire. In these practical measurements, only the optical sensor HRM-2511E was placed inside the MRI scanning area. The Easy Pulse V1.1 module together with the power bank supply and the audio mixer were located outside the shielding cage [23] of the MRI equipment in the control room near the operating console. The position of the tested person was chosen in such a way that the head was placed in the RF scan coil between the upper and lower gradient coils of the MRI device [18,19,20,23] to maximize the noise and vibration effect on the examined person.

Depending on the applied types of stimuli in the *Load* phases, a different number of volunteer healthy persons took part in our experiments with measurement of the BP and HR values, and simultaneous recording of the PPG signal. In auxiliary experiments, 10 persons (7 males and 3 females) were tested with the age ranging from 26 to 86 years. In accordance with the designed measuring protocol in Figure 3, four data records per person were always collected, 40 in total. As mentioned in Introduction, reactions to the applied physiological stimuli are very individual. The tested persons must be taken into consideration (especially in the case of the seniors) together with the experimental possibility depending on the place (performed measurements in the lab environment for younger tested persons and in the home environment for older ones), as well as their physical condition and the age. Table 1 describes briefly our most used activation stimuli depending on the location of the performed experiments. In the case of the measurement inside the MRI device, during the scanning process, no movement of the examined person is appropriate because the final MR image can be blurred. The stimulation (load) effect acting on the person is represented by his/her exposition to vibration and acoustic noise subsequently generated by the MRI gradient system.

In the preliminary measurements with the listening of the MRI noise, six persons (three males and three females) took part with the age between 26 and 58 years. In the main measuring experiments inside the MRI device, four volunteers (2 + 2) were examined with the mean age of 46 ± 10 years and weight (affecting the MRI vibration and noise frequency spectrum) from 50 to 80 kg. In all cases, the tested persons were non-smokers. The small group of persons engaged in our preliminary measurements was the same as the group engaged in the main experiments inside the MRI device. This group was a subset of all volunteers who were measured within the auxiliary experiments comprising the PPG signal recording in chosen different physiological as well as psychological activities.

## 5. Experimental Results

**Figure 4:**
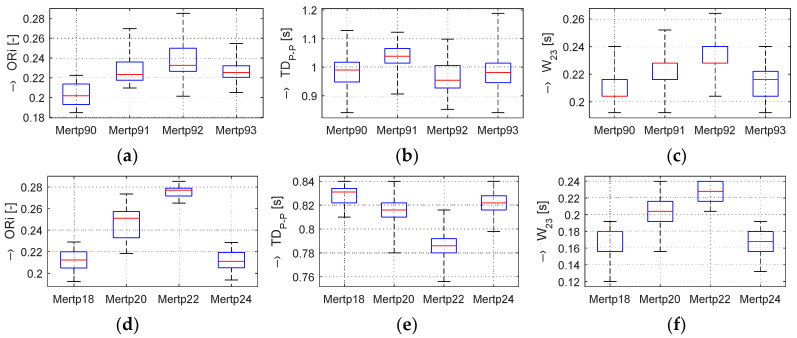
Box-plot of basic statistical parameters of (**a**,**d**) ORi, (**b**,**e**) *TD*_P-P_, and (**c**,**f**) *W*_23_ values determined from PPG signals of male (upper set of three graphs) and female (bottom three graphs) persons in Relax (Mertp90,93 + Mertp18,24) and Load (Mertp91,92 + Mertp20,22) phases.

In the frame of our experiments, the ORi, HR, and BP parameters calculated from the pre-processed PPG signal were analyzed; the determined changes were quantified; and the obtained results were visualized and numerically compared. From the principal point of view, the changes in the calculated ORi are inversely proportional to the period *TD*_P-P_ between the peaks of the systolic blood pressure, as demonstrated by the box-plot of basic statistical parameters in Figure 4. From the literature [14,16], it follows that the typical ORi range lies in the interval of <0.1, 0.3> for healthy people in a normal physiological state. Principally, three possible relations between *TD*_P-P_ and *W*_23_ parameters can practically occur:

1. *TD*_P-P_ is changed, but *W*_23_ is stable,
2. *TD*_P-P_ is stable, but *W*_23_ is changed,
3. *TD*_P-P_ and *W*_23_ are both changed.

The graphs in Figure 4b,e show that, in the Load (Mertp91,92/Mertp20,22) phases, the HR is increasing (the *TD*_P-P_ is shortening) and/or the systolic pulse is widened—see the *W*_23_ parameter in Figure 4c,f. The final effect represents an enhancement of the ORi parameter—it is practically valid for all tested male and female persons.

**Figure 5:**
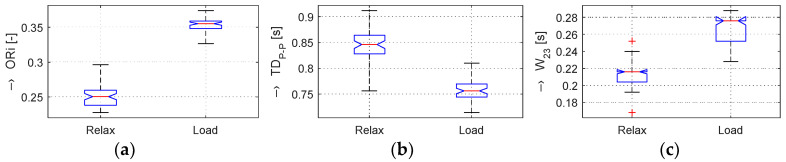

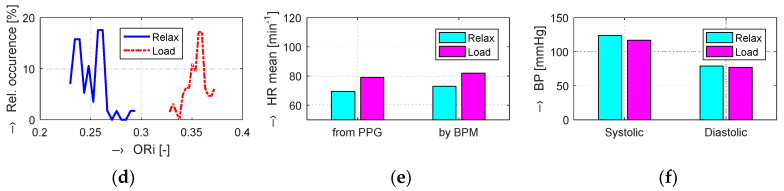
Visualization of changes in evaluated human cardiovascular parameters determined in Relax and Load phases with raising HR and slightly falling BP values: box-plot of basic statistical parameters of (**a**) ORi, (**b**) *TD*_P-P_, and (**c**) *W*_23_ values; (**d**) comparison of histograms of ORi values; (**e**) bar-graphs of HR determined from the PPG signal and measured by the BPM device; (**f**) comparison of systolic and diastolic blood pressure values; male person.

**Figure 6:**
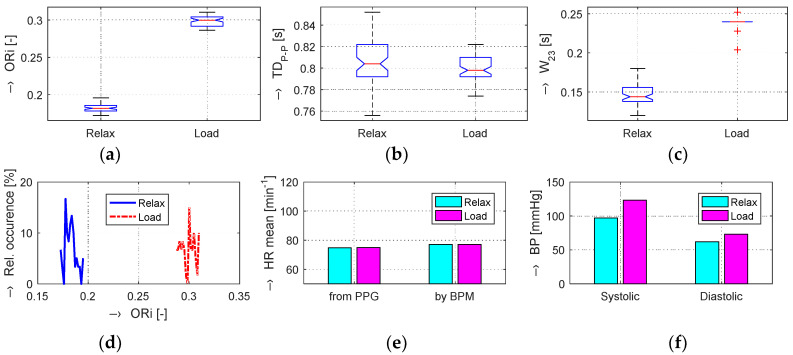
Changes in evaluated human cardiovascular parameters determined in Relax and Load phases with raising BP values and practically unchanged HR values: box-plot of basic statistical parameters of (**a**) ORi, (**b**) *TD*_P-P_, and (**c**) *W*_23_ values; (**d**) comparison of histograms of ORi values; (**e**) bar-graphs of HR determined from the PPG signal and measured by the BPM device; (**f**) comparison of systolic and diastolic blood pressure values; female person.

**Figure 7:**
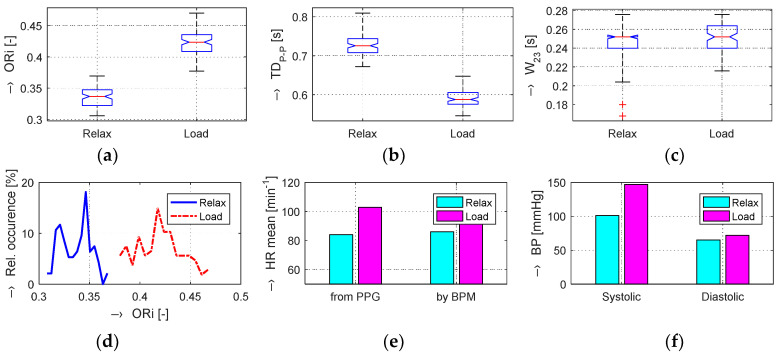
Changes in evaluated human cardiovascular parameters determined in relaxation and sporting stimulation phases with both BP and HR values significantly increased: box-plot of basic statistical parameters of (**a**) ORi, (**b**) *TD*_P-P_, and (**c**) *W*_23_ values; (**d**) comparison of histograms of ORi values; (**e**) bar-graphs of HR determined from the PPG signal and measured by the BPM device; (**f**) comparison of systolic and diastolic blood pressure values; male person.

**Figure 8:**
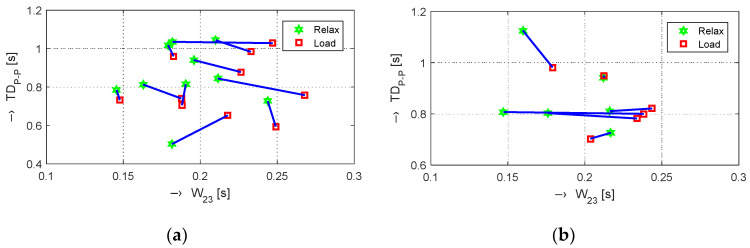
Visualization of mutual positions of *TD*_P-P_ and *W*_23_ parameters—compare with the examples of graphs in Figure 5, Figure 6 and Figure 7b,c determined from histograms in *Relax* and *Load* phases for (**a**) male and (**b**) female persons.

**Figure 9:**
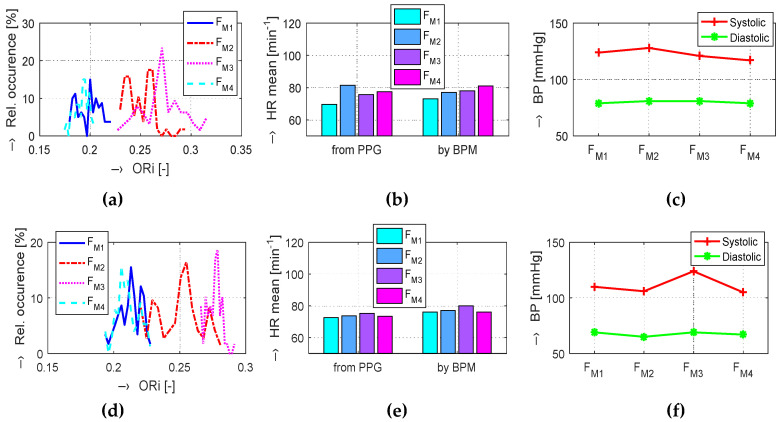
Visualization of changes of cardiovascular parameters determined in the initialization/relaxation (F_M1_, F_M4_), and after the stimulation phase while hearing the MRI noise through headphones (F_M2_, F_M3_), for a male (upper graphs) and a female (bottom graphs) tested person: (**a**,**d**) comparison of histograms of ORi values, (**b**,**e**) bar-graphs of HR determined from the PPG signal and measured by the BPM device, (**c**,**f**) comparison of systolic and diastolic blood pressure values.

**Figure 10:**
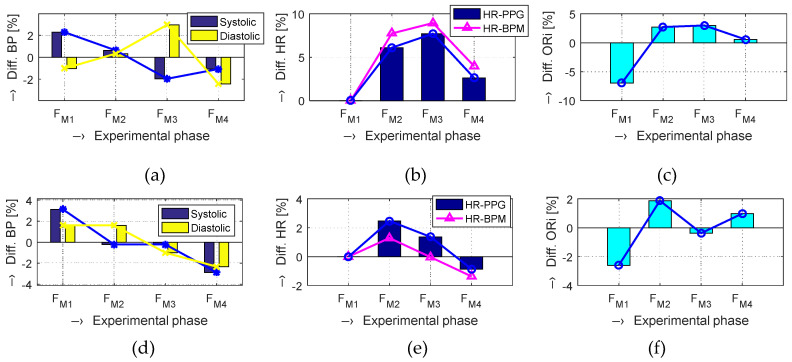
Comparison of differential values in [%] measured by the BPM device and determined from the PPG signal for a male (upper graphs) and female (bottom graphs) tested person lying inside the magnetic resonance imaging (MRI) device during execution of the MR scan sequence: (**a**,**d**) BP values in four measuring phases; (**b**,**e**) HR values determined from PPG and measured by BPM, related to the F_M1_ as a baseline; (**c**,**f**) mean ORi values (after LT removal).

The obtained results are structured by three basic types of experiments mentioned in the previous paragraphs. This means that the sets of graphs in Figure 5, Figure 6 and Figure 7 represent the results for basic statistical parameters of ORi, *TD*_P-P_, and *W*_23_ values, together with differences of HR and ORi values determined from PPG signals recorded in relaxation as well as stimulation phases together with manually measured values of BP. The main results of the ORi parameter were then divided according to rise/fall of the measured HR or the systolic BP. The summary results—mean and standard deviation (std) of percentage differences between values in relaxation and stimulation phases depending on the gender of all tested persons during auxiliary and preliminary experiments—can be seen in Table 2. The graphical comparison of mutual positions of *TD*_P-P_ and *W*_23_ parameters determined in the Relax and Load phases is presented in Figure 8. Figure 9 shows the visualization of changes of these parameters in the stimulation phase while hearing the MRI noise through headphones. Finally, the graphical comparison of the obtained differences measured directly in the scanning area of the MRI device is shown in Figure 10 and the numerical evaluation of all three parameters of the human cardiovascular system is summarized in Table 3, Table 4 and Table 5.

## 6. Discussion of Obtained Results

Although the heart rate variability affected by the stress has been examined by other authors, the use of the ORi feature from the measured fingertip PPG was not found in the scientific literature of the current period, so it can be treated as a new approach. As an example, other pulse wave features (the crest time, the duration of diastole, and the inflection point area ratio) were extracted from the arm PPG [26]. The limitation of our presented work lies, first of all, in the fact that measurement and PPG signal recording experiments inside of the MRI device were applied on a small group of tested persons. In this stage of our research, only six volunteer persons (healthy people, colleagues and authors themselves) took part in the current experiments, so it is very difficult to obtain results having good statistical credibility. This resulted primarily in a great variation of the results (see std values in Table 2 and Table 3 and box-plot graphs in Figure 4). For this reason, only basic statistical parameters were calculated. This further means that the obtained results cannot be generalized, only special as well as typical cases that occurred during our experiments can be described and discussed. The possible solution to this issue may be cooperation with some medical center (in Bratislava, Brno, Vienna, and so on) having a certificate for the work with patients. Both of the MRI devices working with low magnetic field located at our institute can be used for non-clinical and non-medical research only [18,19,20].

The currently performed experiments have shown that physiological stimulation in the *Load* phases produces changes in the ORi values owing to the increased HR or/and the increased systolic BP. In two cases, the changes of BP values for both of the tested phases are similar and the changes of HR ones are different (typically increasing in the Load phase) or, sometimes, there is a synergy effect in the changes of these parameters (most often, HR and BP are increased in the Load phase). In one case of a female person tested during measurements inside the MRI device, the HR and BP changed in the opposite manner (HR increasing, BP decreasing)—this was probably caused by her adaption to the changed position (from staying to lying) and, at the same time, being rather nervous because of the location in a foreign environment inside the MRI scanning area precepted as somewhat unfriendly. The phenomenon of decreased systolic as well as diastolic BP and increased HR after exercises has also been observed in [27]. The second untypical case occurs when the BP and HR values of a tested person lying inside the MRI device were continually falling. After the experiment, this person admitted that she had more felt the vibration to be rather monotonous than to be affected by a stress (this person had previous experience with scanning in MRI devices, so this could have also influence on her lower sensitivity to this type of stimuli).

At this research stage, we can say that, in the majority of the performed measurements, the final obtained values of the ORi parameter are higher than the ORi values determined from the PPG signal recorded during the Relax phases (see summarized mean absolute and differential ORi values in Table 3). The same effect, although not so pronounced, was observed for the audio stimuli applied by the recorded MRI noise and for measuring inside the MRI device with the running MR scan sequence, as documented by the results in graphical as well as numerical form.

The detailed analysis of the obtained BP values presented in Table 4 shows higher systolic pressure at the start and its continuous fall during the experiment for the female persons. On the other hand, the group of the male tested persons exhibited raised BP values only in the second and third stimulation phases and lower systolic as well as diastolic BPs in the beginning and ending relaxation phases. This might be caused by higher stress of more sensible female persons before the experiment during positioning in the scanning area (e.g., a constrained space inside a closed metal cage). Distribution of the merged male and female results is more even, as can be seen in the last line of Table 4.

The numerical comparison of HR values from the PPG signal and from the BPM device means differential mean values related to the *F*_M1_ as a baseline are similar (see Table 5). The maximum HR rise of about 8% (5% in the case of BPM measurements) was achieved for the female persons in the second *Load F*_M3_ phase and 6.5% (5.1%, respectively) in the final *Relax F*_M3_ phase. On the other hand, the results of the male tested persons exhibit maximum values (slightly over 3%) in the first Load phase *F*_M2_. In the last Relax phase *F*_M4_, the HR differential values (0.8% or 1.7% for BPM measurement) are significantly lower than the female ones. The merged results of all tested persons show the maximum increase of HR values for the *F*_M3_ phase using both measuring methods.

## 7. Conclusions

The current article is practically an extension of our previous work [25], where our first experiments with sensing and analyzing of a PPG signal have been described. The choice of the optical sensor HRM-2511E working in transmission mode together with the analog interface Easy Pulse was motivated by its successful application in the measurement that was already published in [25]. Prior to it, we had also tested several PPG sensors based on a reflection principle, but the obtained results were unsatisfactory owing to not very good PPG signal purity and stability. Although relatively few measurements and comparative experiments on a small group of tested persons were made, our working premise about invocation of stress factors by different physiological and psychological stimuli showing changes in ORi, HR, and BP parameters was successfully verified. The performed experiments confirm that the proposed method and the used type of instrumentation for continual automatic sensing of the PPG signal make it possible to detect changes of HR and mainly ORi accompanied by variations in the blood pressure measured manually in discrete time intervals by the portable BPM device.

In a practical realization of all recording experiments, the PPG signal was always picked up from the pinkie of the right hand and the cuff was put on the left arm to prevent any pressure effect on the venous system during the measurement by the BPM device. For confirmation and quantification of this effect, further measurement and comparison must be performed. Therefore, we plan to carry out simultaneous acquisition of the PPG signal and measurement of the BP and HR parameters on the same hand by a portable BPM device with a detailed time analysis of the measured pressure. In addition, we will evaluate PPG signals acquired from different positions of a sensor on a human body [28]. In our case, first of all, we will test sensing the PPG signal from all the fingers (from a pinkie to a thumb) of the left and right hands, with the final aim to verify a possibility of application of a general recommendation for all tested persons, or to confirm a necessity to use individual settings for every person.

Because the Oliva–Roztocil index can also be used as an approach to pain quantification [15,16], we will try to use this parameter for the testing and monitoring of possible pain of people wearing some ortheses, exoskeletons, or other rehabilitation aids during scanning inside an open-air as well as a whole-body MRI device. Generally, examined persons inside a tube of a whole-body tomograph feel more stress and discomfort than in the case of an open-air type; furthermore, the generated vibration and acoustic noise are higher. Our current research was performed using the open-air MRI device only for technical reasons. It is not practically possible to place the PPG sensor including its power supply and cable connection to the recording laptop inside a tube of the closed whole-body tomograph without any disturbance of the homogeneity of the working magnetic field. A similar placement problem exists with an external portable BPM device, where the rubber tube from the pressure cuff on the arm does not have sufficient length to reach outside the scanning area of the closed MRI machine. In addition, the currently used analog interface for the PPG signal recording via long cables from the shielding cage of the open-air MRI device to the mixer with A/D convertor causes many complications and limitations, as well as discomfort for the tested person and for the examiner controlling the whole experiment. Therefore, we would like to develop a new wearable PPG sensor enabling wireless connection [29] for direct data transfer to the recording PC, laptop, tablet, smartphone, or other proper device suitable for transmission via the Bluetooth connection standard. For measurement in the low magnetic field environment (in the scanning area of the MRI device), the sensor must be constructed without any ferromagnetic materials and should be shielded to prevent the influence of RF impulses. This solution will also enable investigation into the scanning tube of the whole-body MRI equipment.

## Figures and Tables

**Table 1:** Brief synopsis of the used types of activation stimuli depending on the experiment location and the age group of tested persons.

Place of Experiment/Age Group of Person	Types of Activation Stimuli
Office (lab)/ Young and Adults	{running in the corridor or upstairs, indoor exercising (sporting), listening to hard music, drinking of a hot beverage (coffee or tea), and so on}
Home environment/Seniors	{cooking, vacuum-cleaning, floor sweeping, a little house work, watching TV political debates, and so on}

**Table 2:** Mean (std in parentheses) values of relative differences in [%] of blood pressure (BP), heart rate (HR), and Oliva–Roztocil index (ORi) parameters between Relax and Load phases obtained from auxiliary and preliminary experiments. BPM, BP monitor.

Parameter/Subject Type	Mean [%] (std)		
	Male	Female	Male + Female
BP systolic	0.39 (0.17)	2.24 (1.26)	0.59 (0.53)
BP diastolic	−1.52 (0.74)	−0.99 (1.07)	−1.32 (0.84)
HR by BPM	5.62 (0.71)	5.72 (0.66)	5.66 (067)
HR from PPG	7.48 (0.86)	5.99 (0.76)	6.92 (0.81)
ORi	14.29 (1.65)	20.28 (2.05)	16.53 (1.76)

**Table 3:** Comparison of mean absolute ORi (std in parentheses) values of and mean differential ORi values for persons examined in the magnetic resonance imaging (MRI) device.

Subject Type	Abs. ORi [–]				Diff. ORi ^(1)^ [%]			
	*F*_M1_	*F*_M2_	*F*_M3_	*F*_M4_	*F*_M1_	*F*_M2_	*F*_M3_	*F*_M4_
Male	0.232 (0.048)	0.253 (0.063)	0.310 (0.094)	0.262 (0.074)	−6.79	−2.15	10.05	−1.10
Female	0.243 (0.061)	0.247 (0.051)	0.266 (0.047)	0.232 (0.034)	−2.69	−0.07	5.84	−3.07
Male+female	0.236 (0.053)	0.251 (0.059)	0.296 (0.084)	0.251 (0.064)	−5.25	−1.37	8.47	−1.84

^(1)^ After LT removal.

**Table 4:** Mean differential values of BP parameters determined in relaxation (*F*_M1,4_) and stimulation (*F*_M2,3_) phases for persons examined in the MRI device.

Subject Type ^(1)^	Diff. BP Systolic [%]				Diff. BP Diastolic [%]			
	*F*_M1_	*F*_M2_	*F*_M3_	*F*_M4_	*F*_M1_	*F*_M2_	*F*_M3_	*F*_M4_
Male	−0.59	3.36	0.08	−2.85	−0.26	0.25	−0.72	0.73
Female	3.13	0.81	−0.08	−3.86	3.92	−0.96	−1.15	−1.81
Male+female	0.81	2.40	0.02	−3.23	1.3079	−0.20	−0.88	−0.22

^(1)^ Used hi-res SE-HF scan sequences with TE = 18 ms, TR = 400 ms, and sagittal orientation.

**Table 5:** Mean differential values of HR values determined from PPG and measured by BPM in relaxation (*F*_M1,4_) and stimulation (*F*_M2,3_) phases for persons examined in the MRI device.

Subject Type	Diff. HR ^(1)^ from PPG [%]				Diff. HR ^(1)^ by BPM [%]			
	*F*_M1_	*F*_M2_	*F*_M3_	*F*_M4_	*F*_M1_	*F*_M2_	*F*_M3_	*F*_M4_
Male	0	3.23	3.19	0.76	0	3.13	2.94	1.71
Female	0	4.14	7.79	6.48	0	2.86	5.31	5.07
Male + female	0	3.57	4.92	2.89	0	3.03	3.83	2.97

^(1)^ Related to the *F*_M1_ as a baseline.
